# 591. The Agency for Healthcare Research and Quality (AHRQ) Safety Program for MRSA Prevention: Impact of an Educational and Implementation Project in United States (US) Hospitals

**DOI:** 10.1093/ofid/ofae631.186

**Published:** 2025-01-29

**Authors:** Lisa L Maragakis, Melissa A Miller, Leyi Lin, Roy Ahn, Kathleen Speck, Yue Gao, Jennifer Titus, Prashila Dullabh

**Affiliations:** Johns Hopkins Medicine, Baltimore, MD; Agency for Healthcare Research and Quality, Rockville, Maryland; Agency for Healthcare Research and Quality, Rockville, Maryland; NORC, Chicago, Illinois; Johns Hopkins, Baltimore, Maryland; NORC, Chicago, Illinois; NORC at the University of Chicago, West Kill, New York; NORC at the University of Chicago, West Kill, New York

## Abstract

**Background:**

The AHRQ Safety Program for MRSA Prevention utilizes evidence-based infection prevention interventions and the Comprehensive Unit-based Safety Program (CUSP) framework to decrease invasive methicillin-resistant *Staphylococcus aureus* (MRSA) infections in intensive care units (ICUs), non-ICUs, surgical services, and long-term care facilities. We report the program's effect on hospital-onset MRSA bacteremia rates (HOB-MRSA) in 106 ICUs and 87 non-ICUs from 94 US hospitals.

**Table 1.**
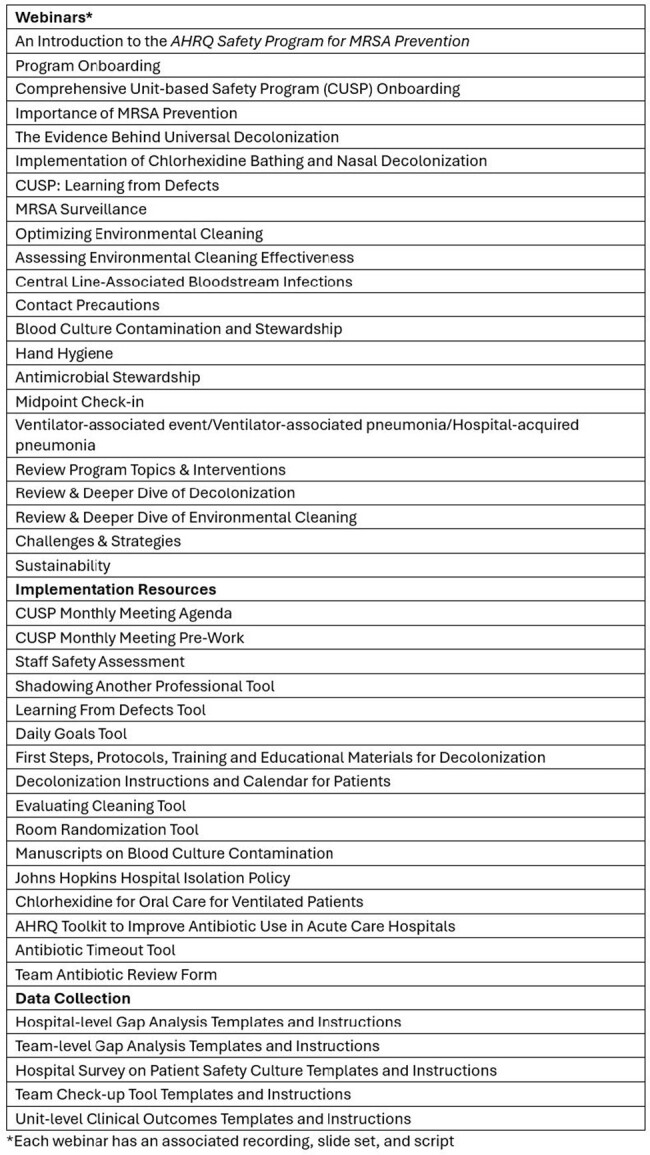
Educational Toolkit Content for the AHRQ Safety Program for MRSA Prevention

**Methods:**

The ICU/non-ICU Safety Program for MRSA Prevention was implemented from April 2022 to September 2023. The Safety Program aimed to decrease invasive MRSA infections through education, technical support, reinforcement of safety culture, and implementation of evidence-based infection prevention practices. Chlorhexidine bathing and nasal decolonization were major foci of the intervention, along with disinfection of the environment and practices to prevent person-based MRSA transmission and device-related infections. The project team provided 22 live webinars, supporting materials, and other tools to assist units with MRSA prevention (Table 1). Units were also assigned an implementation adviser who provided support through monthly coaching calls.

Units submitted monthly data for HOB-MRSA per 10,000 patient days (PD) as well as data regarding process measures. Linear mixed effects models were employed to calculate pre-post intervention changes in HOB-MRSA events.

**Figure:**
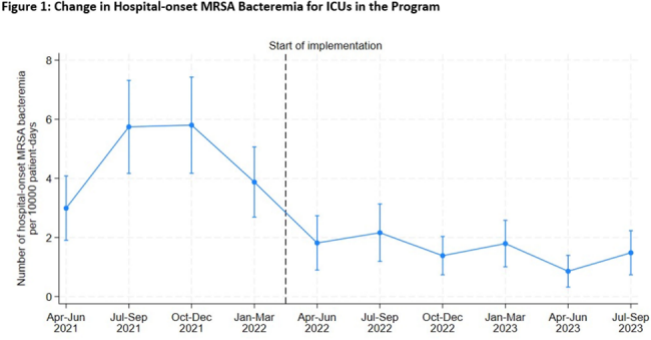

**Results:**

106 ICUs and 87 non-ICUs from 94 hospitals completed the Safety Program, including 31 (33%) academic medical centers (AMC), 37 (39%) non-AMC teaching hospitals, and 26 (28%) non-teaching community/other hospitals. From July-September 2021 to July-September 2023, HOB-MRSA events declined from 5.7 to 1.5 per 10,000 PD among ICUs (p< 0.001)(Figure 1) and from 3.4 to 0.2 for non-ICUs (p< 0.001) (Figure 2).

Similar results were found in comparisons among other pre-program vs, end-of-program quarters.

**Figure:**
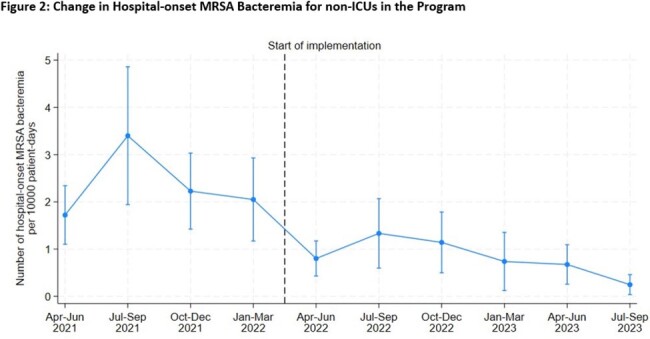

**Conclusion:**

The AHRQ Safety Program for MRSA Prevention supported implementation of evidence-based infection prevention practices, including decolonization, and was associated with reduced HOB-MRSA rates across participating ICUs and non-ICUs.

**Disclosures:**

**All Authors**: No reported disclosures

